# Small Molecule ErbB Inhibitors Decrease Proliferative Signaling and Promote Apoptosis in Philadelphia Chromosome–Positive Acute Lymphoblastic Leukemia

**DOI:** 10.1371/journal.pone.0070608

**Published:** 2013-08-01

**Authors:** Mary E. Irwin, Laura D. Nelson, Janice M. Santiago-O’Farrill, Phillip D. Knouse, Claudia P. Miller, Shana L. Palla, Doris R. Siwak, Gordon B. Mills, Zeev Estrov, Shulin Li, Steven M. Kornblau, Dennis P. Hughes, Joya Chandra

**Affiliations:** 1 Department of Pediatrics Research, The University of Texas MD Anderson Cancer Center, Houston, Texas, United States of America; 2 Department of Systems Biology, The University of Texas MD Anderson Cancer Center, Houston, Texas, United States of America; 3 Department of Biostatistics, The University of Texas MD Anderson Cancer Center, Houston, Texas, United States of America; 4 Department of Leukemia, The University of Texas MD Anderson Cancer Center, Houston, Texas, United States of America; 5 The University of Texas Graduate School of Biomedical Sciences, Houston, Texas, United States of America; University of South Alabama, United States of America

## Abstract

The presence of the Philadelphia chromosome in patients with acute lymphoblastic leukemia (Ph^+^ALL) is a negative prognostic indicator. Tyrosine kinase inhibitors (TKI) that target BCR/ABL, such as imatinib, have improved treatment of Ph^+^ALL and are generally incorporated into induction regimens. This approach has improved clinical responses, but molecular remissions are seen in less than 50% of patients leaving few treatment options in the event of relapse. Thus, identification of additional targets for therapeutic intervention has potential to improve outcomes for Ph+ALL. The human epidermal growth factor receptor 2 (ErbB2) is expressed in ∼30% of B-ALLs, and numerous small molecule inhibitors are available to prevent its activation. We analyzed a cohort of 129 ALL patient samples using reverse phase protein array (RPPA) with ErbB2 and phospho-ErbB2 antibodies and found that activity of ErbB2 was elevated in 56% of Ph^+^ALL as compared to just 4.8% of Ph^−^ALL. In two human Ph+ALL cell lines, inhibition of ErbB kinase activity with canertinib resulted in a dose-dependent decrease in the phosphorylation of an ErbB kinase signaling target p70S6-kinase T389 (by 60% in Z119 and 39% in Z181 cells at 3 µM). Downstream, phosphorylation of S6-kinase was also diminished in both cell lines in a dose-dependent manner (by 91% in both cell lines at 3 µM). Canertinib treatment increased expression of the pro-apoptotic protein Bim by as much as 144% in Z119 cells and 49% in Z181 cells, and further produced caspase-3 activation and consequent apoptotic cell death. Both canertinib and the FDA-approved ErbB1/2-directed TKI lapatinib abrogated proliferation and increased sensitivity to BCR/ABL-directed TKIs at clinically relevant doses. Our results suggest that ErbB signaling is an additional molecular target in Ph^+^ALL and encourage the development of clinical strategies combining ErbB and BCR/ABL kinase inhibitors for this subset of ALL patients.

## Introduction

The Philadelphia chromosome (Ph), is present in ∼5% of pediatric and 30% of adult cases of acute lymphoblastic leukemia (ALL) [1]. Ph^+^ALL is the most aggressive subtype of ALL [2]. Since 2001, when imatinib, a BCR/ABL-directed small molecule tyrosine kinase inhibitor (TKI), was approved for clinical use, response rates have improved for patients with this chromosomal translocation [1]. Unfortunately, hematologic response rates to imatinib are worse in Ph^+^ALL than in chronic myelogenous leukemia (CML) [1]. Clinically, combinations with chemotherapy and second generation BCR/ABL-directed TKI have improved response rates, however, due to resistance and inevitable relapse, the average overall survival remains near 50% [1]. Due to this relative lack of efficacy, discovery of new therapeutic targets is imperative for the treatment of this leukemia subtype.

The ErbB receptor tyrosine kinase family is expressed in many different cancer types where it promotes survival and proliferative signaling. This strong link to the oncogenic phenotype led to the therapeutic targeting of ErbB receptors with a variety of compounds. One family member, ErbB2 is expressed within B-lymphoid blast cells from patients with ALL and CML [3], [4]; however, these studies did not examine ErbB2 expression or activity across ALL subtypes including Ph^+^ALL. Because of its relationship with growth and survival signaling, we sought to determine whether this protein family could be a novel target in the treatment of Ph^+^ALL. Using reverse phase protein array (RPPA) analyses, we show that Ph^+^ALL patients have higher expression of phospho-ErbB2 compared to Ph^−^ALL, and that the ErbB kinase inhibitors canertinib and lapatinib abrogate proliferative signaling while promoting apoptotic signaling. We document caspase-dependent cell death in patient derived Ph^+^ALL lines after treatment with ErbB TKIs alone and in combination with BCR/ABL-directed TKI, providing impetus for the clinical testing of this strategy for ErbB2-expressing Ph^+^ALL.

## Materials and Methods

### Cell Lines and Reagents

Human Ph^+^ALL cell lines, Z181 and Z119 [5], were cultured at 5% CO_2_ in RPMI-1640 medium containing 10% fetal bovine serum (Gibco, Grand Island, NY), 1% penicillin/streptomycin, and 1% L-glutamine. Canertinib was received from Pfizer, Inc. (New York, NY) and lapatinib, imatinib, nilotinib, and dasatinib were purchased from LC Laboratories (Woburn, MA).

### Patient Population

Peripheral blood and bone marrow specimens were collected from 129 adult patients with newly diagnosed ALL evaluated at The University of Texas M.D. Anderson Cancer Center (MDACC) between 1992 and May 2007. Samples were acquired during routine diagnostic assessments in accordance with the regulations and protocols (Lab 01-473) approved by the Investigational Review Board (IRB) of the University of Texas MD Anderson Cancer Center. Written informed consent was obtained in accordance with Declaration of Helinski. Samples were analyzed under an IRB-approved laboratory protocol (Lab05-0654). The median age of these patients was 39.7 years (range 15–80). Cytogenetics of the population include 35 diploid, 18 hyperdiploid, 8 hypodiploid, 12 pseudodiploid, 5 insufficient metaphases (IM), 5 no analyzable metaphases (NAM), 8 miscellaneous, 25 Ph^+^, 2 t(11;14), 7 t(4;11), 2 t(8,14), 1 t(8;22), and 1 t(11;19). The samples were normalized to a concentration of 1×10^4^ cells/µL and a whole cell lysate prepared as previously described [6].

### RPPA Analysis

The methodology and validation of RPPA are fully described in previous publications [7], [8], [9]. Briefly, patient samples were printed in 5 serial dilutions onto slides along with normalization and expression controls. Slides were probed with strictly validated primary antibody and a secondary antibody to amplify the signal, and finally a stable dye [10] was precipitated. The stained slides were analyzed using Microvigene® software (Vigene Tech, Carlisle, MA) to produce quantified data.

### Western Blotting

Cell lysates were prepared using Triton X-100 buffer (phosphate buffered saline (PBS) with 1% Triton X-100; 25 mM Tris, pH 7.5; and 150 mM NaCl) containing a protease inhibitor cocktail (Roche, Indianapolis, IN) and phosphatase inhibitor cocktail 2 (Sigma Aldrich, St. Louis, MO). Proteins were separated by sodium dodecyl sulfate (SDS)-polyacrylamide gel electrophoresis (PAGE) and detected by Western Blot utilizing antibodies specific to ErbB2 (Cell Signaling Technology, Danvers, MA), ErbB2 Y1248p (Millipore, Billerica, MA), S6-kinase S240p (Cell Signaling Technology), S6-kinase (Cell Signaling Technology), p70S6-kinase (Epitomics, Burlingame, CA), p70S6-kinase T389p (Cell Signaling Technology), Bim (Epitomics), PARP (kindly provided by Dr. Scott Kaufman, Mayo Clinic, Rochester, MN), and actin (Sigma-Aldrich). Bands were visualized using corresponding secondary antibodies followed by chemiluminescent detection (GE Healthcare, Waukesha, WI). Densitometry was performed using ImageJ software (National Institutes of Health, Bethesda, MD).

### Quantification of Cell Surface ErbB2

One million cells were harvested and resuspended in 50 µL PBS containing 20 µL phycoerythrin (PE)-conjugated ErbB2 (PE-ErbB2) or PE-mouse immunoglobulin G (IgG) antibody (BD Biosciences, San Jose, CA) and incubated for 30 minutes on ice. Bead quantification standard curve was prepared in accordance with the manufacturer’s instructions (Bangs Laboratories, Inc., Fishers, IN). Samples were analyzed on the FL-2 channel of a fluorescence-activated cell sorter (FACSCalibur; Becton Dickinson, Franklin Lakes, NJ) using CellQuest software (Becton Dickinson). Median fluorescence values were quantified and compared with the bead standard curve.

### Caspase-3 Activity

Cells were plated at a density of 1.0×10^6^ cells per milliliter in a 6-well plate and incubated for 24 hours with canertinib (0–5.0 µM). To lyse the cells, 3 freeze/thaw cycles were performed in 1XPBS followed by centrifugation. Samples were adjusted to 1 mg/mL and 50 µL of cell suspension was plated into an opaque 96-well plate. One hundred fifty microliters of 50 µM DEVD-7-amino-4methylcoumarin (AMC) was then added to each sample and incubated for 3 hours in the dark. Samples were analyzed using a SpectraMax Gemini EM plate reader (Molecular Devices, Sunnyvale, CA).

### Subdiploid, Proliferation, and Viability Analyses

Z181 and Z119 cells were plated at a density of 0.5×10^6^ cells/milliliter in a 24-well plate and treated with indicated doses of canertinib, lapatinib, and/or imatinib, nilotinib, or dasatinib for indicated times. Cells were then harvested, washed, then either resuspended in propidium iodide (PI) solution (50 *µ*g/mL PI, 0.1% Triton X-100, and 0.1% sodium citrate in PBS) and incubated for at least 3 hours at 4 degrees and then PI fluorescence was read on the FL-3 channel of the FACSCalibur and analyzed using CellQuest software or cell number and viability were analyzed using trypan blue exclusion via Vi-CELL Cell Viability Analyzer (Beckman Coulter, Brea, CA).

### Statistical Analyses

For RPPA: *Supercurve* algorithms were used to generate a single value from the 5 serial dilutions [11]. Loading control [12] and topographical normalization procedures accounted for protein concentration and background staining variations. Analysis using unbiased clustering, perturbation bootstrap clustering and principle component analysis was then performed as fully described [7]. Association between protein expression levels and categorical clinical variables were assessed in R using standard t tests, linear regression or mixed-effects linear models. Association between continuous variable and protein levels were assessed by using Pearson and Spearman correlation and linear regression. Bonferroni corrections were performed to account for multiple statistical parameters for calculating statistical significance. For non-RPPA: Student *t*-tests were used to compare between treatment groups. A *p*-value of less than 0.05 was considered significant.

## Results

### ErbB2 Protein Expression and Activation are Elevated within the Ph^+^ALL Patient Population

Although previous studies have demonstrated expression of ErbB2 in a subset of patients with B-lineage-ALL and CML in B-lymphoid blast crisis, they have not established whether ErbB2 protein expression or activity was associated with negative prognostic indicators [3], [4]. To determine the incidence of ErbB2 protein overexpression in ALL, RPPA was performed on 129 patient specimens utilizing ErbB2-directed antibodies (Table 1). Elevated or decreased expression was defined as expression levels above or below the 95% confidence interval of CD34+ normal specimen mean expression, respectively. Overexpression of ErbB2 was seen in 28.5% of ALL samples as compared with CD34+ normal specimens. Categorization by cell lineage revealed that 27.4% of B-ALL and 53.3% of T-ALL expressed elevated ErbB2 protein. As Ph-positivity is a negative prognostic indicator in ALL, samples were also stratified by Ph-status. Forty-percent of Ph^+^ALL samples had overexpression of ErbB2 compared to just 27.9% of Ph^−^ALL; however this difference was not statistically significant (p = 0.9362). High ErbB2 positivity was not indicative of elevated ErbB2 activity (as measured by ErbB2 auto-phosphorylation, Table 2) as only 15% of ALL samples had greater than normal ErbB2p. There was also no elevation of ErbB2p in T-ALL, despite the high level of protein expression of ErbB2. However, 56% of Ph^+^ALL samples contained significantly elevated ErbB2p compared to 4.8% of Ph^−^ALL (p<0.0001).

**Table 1:** Total ErbB2 protein expression in ALL patient samples.

	All ALL	T-ALL	B-ALL	Ph^+^ALL	Ph^−^ALL	CD34+
mean	0.1	0.8	0.0	0.0	0.1	0.1
SD	1.7	2.2	1.4	1.7	1.8	0.5
median	0.0	0.8	0.0	0.1	0.0	0.1
min	−4.3	−2	−4.3	−3.8	−4.3	−0.8
max	5.1	4.4	5.1	2.8	5.1	1.7
>norm (%)	28.5	53.3	27.4	40.0	27.9	4.8
<norm (%)	35.0	53.3	33.6	32.0	34.6	4.8
= norm (%)	36.5	13.3	38.9	28.0	37.5	90.5
Obs Num	129	15	114	25	104	21

Log_2_ expression values were median centered (median of CD34+ = 0). All ALL: combined statistics with no subgroup analysis; T-ALL: T-cell ALL samples; B-ALL: B-cell ALL samples; Ph^+^ALL: all ALL samples expressing the Philadelphia chromosome; Ph^−^ALL: samples lacking the Philadelphia chromosome; SD: standard deviation; >norm (%): percentage above values within the 95% confidence interval based on the range of expression of the normal CD34+ cells; <norm(%): percentage below values within the 95% confidence interval based on the range of expression of the normal CD34+ cells; = norm (%): percentage equal to values within the 95% confidence interval based on the range of expression of the normal CD34+ cells; Obs Num: total observed cases per subgroup.

**Table 2:** Phosphorylated ErbB2 expression in ALL patient samples.

	All ALL	T-ALL	B-ALL	Ph^+^ALL	Ph^−^ALL	CD34+
mean	0.0	−0.3	0.00	1.6	−0.4	0.0
SD	1.6	0.8	1.6	1.6	1.2	0.9
median	−0.3	−0.2	−0.4	1.8	−0.6	−0.1
min	−2.6	−0.2	−2.6	−1.8	−2.6	−1.8
max	5.7	1.2	5.7	4.7	5.7	1.7
>norm (%)	15.3	0.0	16.8	56.0	4.8	4.8
<norm (%)	24.1	13.3	24.8	4.0	27.9	4.8
= norm (%)	60.6	86.7	58.4	40.0	67.3	90.5
Obs Num	129	15	114	25	104	21

Log_2_ expression values were median centered (median of CD34+ = 0). All ALL: combined statistics with no subgroup analysis; T-ALL: T-cell ALL samples; B-ALL: B-cell ALL samples; Ph^+^ALL: all ALL samples expressing the Philadelphia chromosome; Ph^−^ALL: samples not containing the Philadelphia chromosome; SD: standard deviation; >norm (%): percentage above values within the 95% confidence interval based on the range of expression of the normal CD34+ cells; <norm(%): percentage below values within the 95% confidence interval based on the range of expression of the normal CD34+ cells; = norm (%): percentage equal to values within the 95% confidence interval based on the range of expression of the normal CD34+ cells; Obs Num: total observed cases per subgroup.

**Figure 1:**
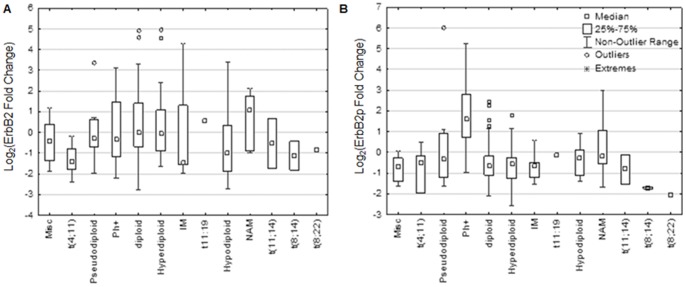
ErbB2 activation, but not expression, varies between ALL cytogenetic categories. RPPA was performed using antibodies directed against ErbB2 (A) and ErbB2p (B) on 129 ALL patient samples and normalized to CD34+ normal cells on a log_2_ scale. Zero indicates the median of CD34+ normal cells. Patient cytogenetics were retroactively analyzed to categorize expression values. Box represents 25–75% of the median population; whiskers represent the range of data. Misc: Miscellaneous, IM: insufficient metaphases, NAM: no analyzable metaphases.

The two primary prognostic indicators in ALL are cytogenetics and Ph-status, however, no particular cytogenetic category was associated with elevated ErbB2 protein expression (Fig. 1A). Ph^+^ALL samples did show higher ErbB2p as compared with all other cytogenetic categories (Fig. 1B). Together, these results suggest that increased activation of ErbB2 is associated moreso with Ph^+^ALL relative to other ALL subgroups.

### Patient-derived Ph^+^ALL Cell Lines Express Targetable Active ErbB2

**Figure 2:**
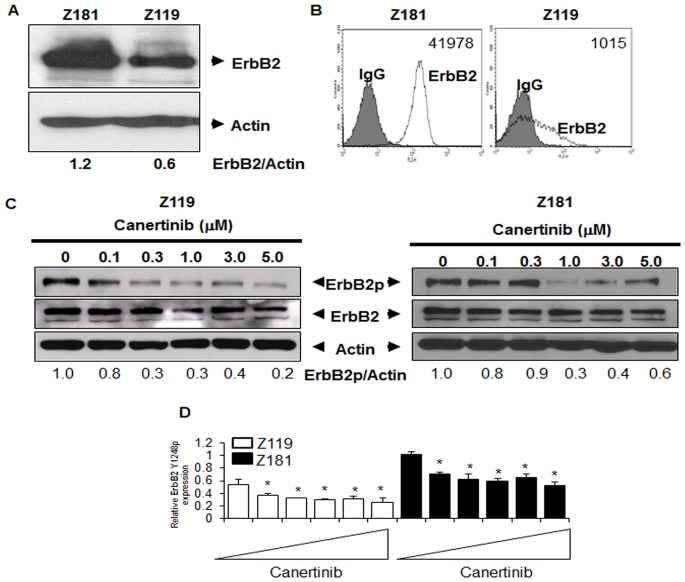
ErbB2 protein expression and activation in Ph^+^ALL cell lines. (A) Two established human Ph^+^ALL cell lines, Z181 and Z119, were lysed and then subjected to SDS-PAGE followed by Western blotting for ErbB2 and actin. Blots are representative of at least three independent experiments. Densitometry was performed using ImageJ (National Institutes of Health). (B) Cell lines were stained with murine PE-conjugated anti-human ErbB2 monoclonal antibody and assessed by flow cytometry; isotype control staining (grey) and anti-ErbB2 staining (white). Cell surface quantification was performed as described in Materials and Methods. Numbers indicate the average number of molecules of ErbB2 per cell. (C) Protein lysates were collected from cells treated with the indicated doses of canertinib for 18 hours. Samples were subjected to SDS-PAGE followed by Western blotting for ErbB2 Y1248p (ErbB2p), total ErbB2 (ErbB2), or actin. Blots are representative of at least three experiments. Densitometry was performed using ImageJ software and normalized to actin. (D) RPPA analyses were performed utilizing ErbB2p antibody. Bars represent the means of three individual experiments. Triangles indicate drug concentrations of 0–5 µM. *p<0.05 compared to untreated.

To confirm a model system of ErbB2^+^Ph^+^ALL, two human-derived Ph^+^ALL cell lines, Z181 and Z119, were lysed and subjected to western blotting (Fig. 2A). Z181 cells contain higher levels of ErbB2 as compared to Z119. Trace expression of ErbB1, ErbB3, and ErbB4 was present in these cell lines (data not shown). ErbB2 acts predominantly at the cell surface to promote signaling [13]. Thus, immunostaining for ErbB2 was performed followed by FACS and enumeration by bead quantification with Mouse IgG staining as a negative control (Fig. 2B). There were 41978±18818 molecules of ErbB2 per Z181 cell and, consistent with our western blotting results, Z119 cells had fewer surface ErbB2 molecules per cell (1015±1312). Together, these data indicate that properly localized ErbB2 is the predominant ErbB isoform present in Ph^+^ALL cell lines.

Since active ErbB2 promotes a myriad of survival and proliferative signaling pathways in cancer cells, the pan-ErbB TKI canertinib was used to block ErbB2 phosphorylation. Western blotting for total- and phospho-ErbB2 (ErbB2p) were performed Z181 and Z119 cells after canertinib treatment (0–5.0 µM) for 18 hours. Canertinib had no effect on total ErbB2 protein levels; however, ErbB2p was significantly decreased in both cell lines (Fig. 2C). RPPA confirmed inhibition of ErbB2p by canertinib under the same conditions (Fig. 2D).

### ErbB-family Kinase Inhibition Affects Survival and Growth Signaling in Ph^+^ALL

**Figure 3:**
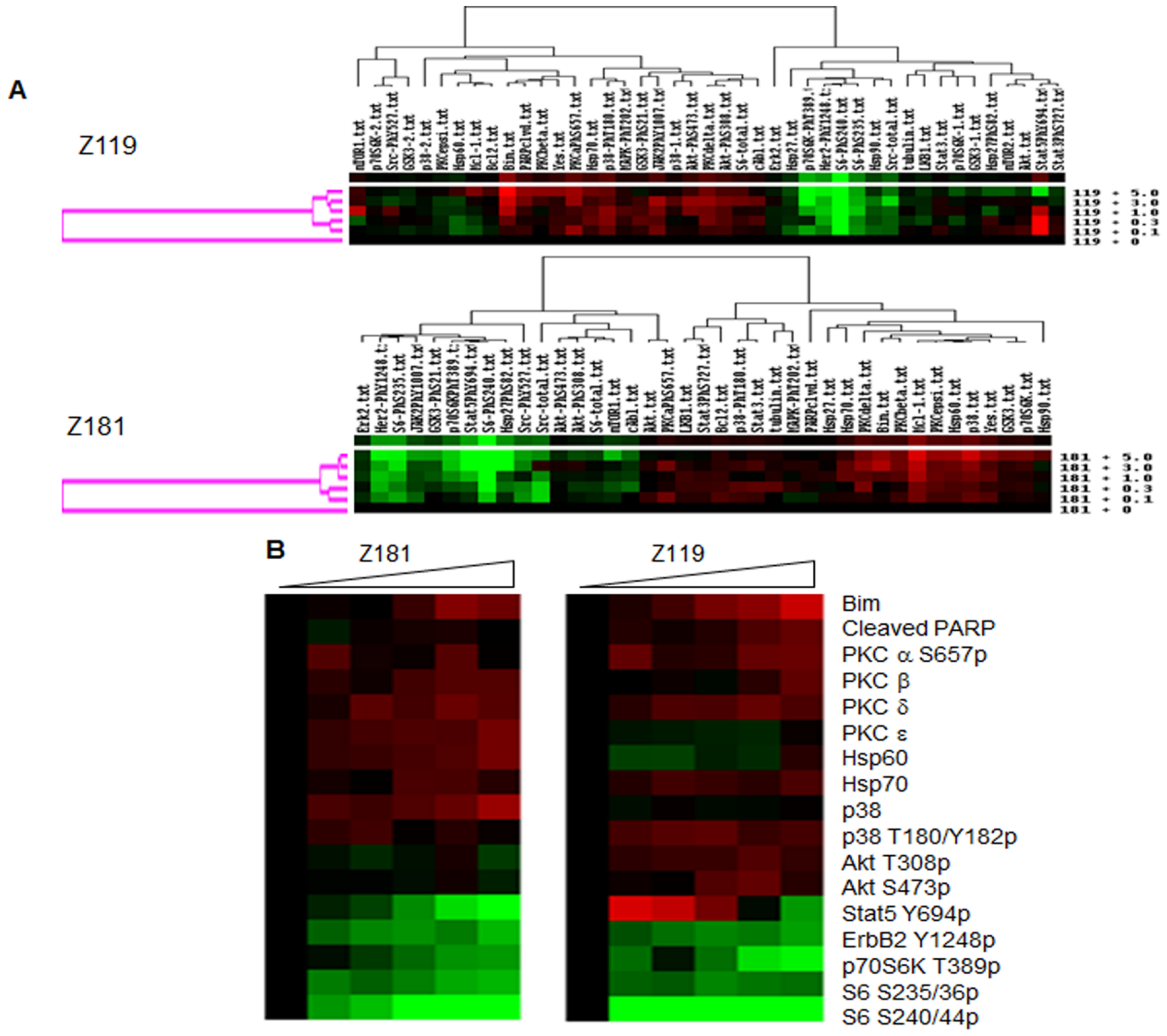
Reverse phase protein analyses of ErbB2^+^Ph^+^ALL with canertinib treatment. (A) Unsupervised clustering analyses were performed on RPPA data from Z181 or Z119 cells treated with 0.1–5 µM of canertinib for 18 hours. To generate heat maps, total protein and phosphoprotein levels were quantified by RPPA, and data were centered on the mean. Intensifying red color indicates increasing protein or phosphoprotein expression relative to the mean, black indicates the mean value, and intensifying green color indicates decreasing levels. (B) Supervised clustering showing relative changes in expression of pro-apoptotic proteins (Bim, cleaved PARP), protein kinase Cs, heat shock proteins, p38 mitogen activated protein kinase, and p38 T180/Y182p; five components of the phosphatidylinositol 3 kinase signaling pathway (Akt T308p, Akt S473p, p70S6-kinase T389p, S6-kinase S235/36p, and S6-kinase S240/44p); and ErbB2 Y1248p in the two cell lines after treatment.

As ErbB receptors are primarily signaling molecules, we sought to determine the effect of ErbB kinase inhibition on downstream pathways. Z181 and Z119 cells were treated with increasing doses of canertinib and RPPA was performed with 43 independently validated antibodies (Table S1). Unsupervised clustering (Pearson correlation; Fig. 3A) and supervised clustering (Fig. 3B) after RPPA analyses showed two distinct groups of responsive proteins in both cell lines: (1) lowered ErbB2 Y1248p along with decreased phosphorylation of p70S6-kinase T389, S6-kinase S235/36, and S6-kinase S240/44, and (2) increased protein kinase C beta, p38 T180p, and pro-apoptotic proteins Bim, cleaved poly ADP-ribose polymerase (PARP), and Mcl-1. The most dramatic responses to canertinib were S6-kinase S240/44p, which was reduced as much as 91% in both cell lines relative to baseline, and S6-kinase S235/36p, which was reduced by 39% in Z119 and 49% in Z181 cells, with no change in total S6-kinase. Similarly, p70S6-kinase T389p was reduced by 66% in Z119 and 35% in Z181 cells (p-values on Table S2). For both cell lines, a cluster breakpoint occurred between 0.3 µM and 1.0 µM canertinib, consistent with an IC_50_ of approximately 1.0 µM.

**Figure 4:**
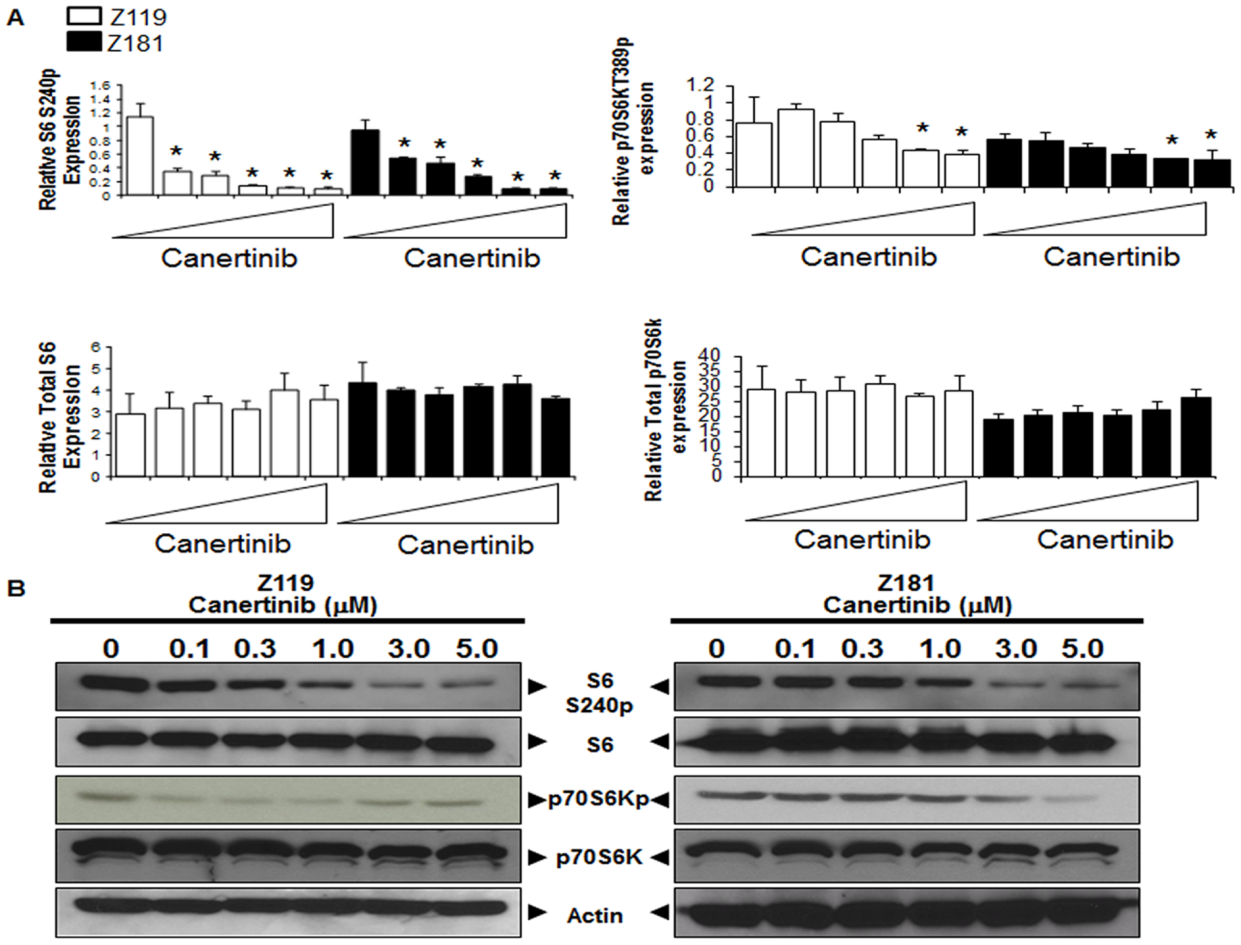
Downregulation of pro-survival signaling induced by canertinib. (A) Protein expression values from RPPA analyses of Z119 (white) and Z181 (black) cells were quantified, and expression relative to the mean graphed. Triangles indicate drug concentrations of 0–5 µM. *p<0.05 compared to untreated. (B) Z119 and Z181 cells were treated with canertinib for 18 hours and then lysed. Samples were subjected to SDS-PAGE followed by immunoblotting with the indicated antibodies.

Western blotting (Fig. 4B) was performed to validate canertinib-induced changes seen in RPPA (Fig. 4A). While total S6-kinase protein levels were unaffected, S6-kinase S240p was reduced by as much as 81% in Z119 and 30% in Z181 after canertinib treatment. Additionally, phosphorylation of p70S6-kinase was reduced while total p70S6-kinase remained unchanged (Fig. 4B). Together, these results agree with those of RPPA and demonstrate that canertinib treatment decreases proliferative and survival signaling.

### ErbB-kinase Inhibition Promotes Apoptosis and Growth Inhibition of Ph^+^ALL Cells

**Figure 5:**
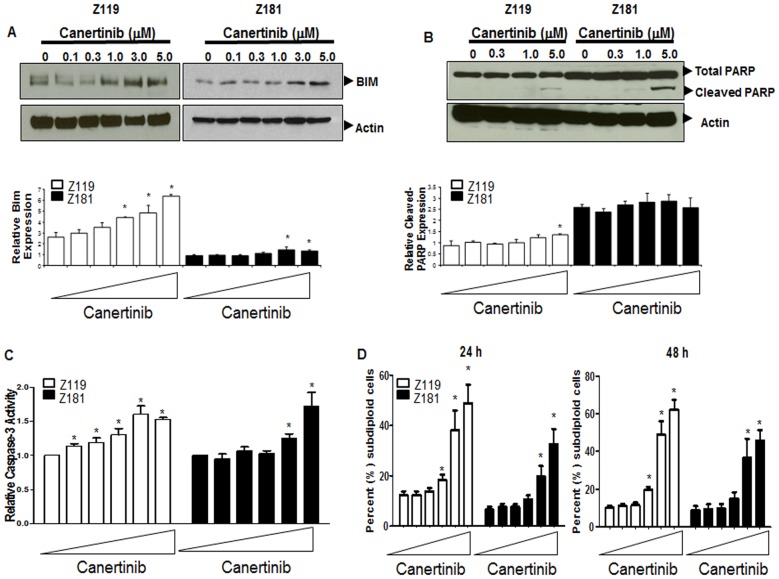
Apoptosis of ErbB2^+^Ph^+^ALL cell lines is induced by canertinib. (A and B, upper panels) Z119 and Z181 cells were treated with canertinib for 18 hours and lysates were subjected to SDS-PAGE followed by immunoblotting with the indicated antibodies. (A and B, lower panels) Protein expression values from RPPA analyses were quantified, and expression relative to the mean graphed. Triangles indicate drug doses of 0–5 µM. *p<0.05 compared to untreated. (C) Caspase-3 activity was assayed after 18 hours of treatment with canertinib using DEVD-AMC fluorogenic substrate. Bars indicate the mean and standard deviation of three independent experiments. *p<0.05 compared to untreated. (D) After treatment with canertinib for either 24 (left panel) or 48 (right panel) hours, cells were stained with PI, and the subdiploid population was quantified by flow cytometry. Bars indicate the mean and standard deviation of at least three independent experiments. *p<0.05. Triangles indicate 0–5 µM doses.

Canertinib treatment also results in the promotion of pro-apoptotic signaling. Most significantly, RPPA showed that Bim was increased by as much as 144% in Z119 and 49% in Z181 cells (Fig. 5A, bar graphs). In concordance with these analyses, western blotting showed that total Bim protein (Fig. 5A) was elevated in a dose-dependent manner after canertinib treatment. While elevations of cleaved-PARP were only seen in Z119 cells by RPPA, western blotting showed PARP cleavage in both cell lines after canertinib treatment (Fig. 5B).

To implicate apoptosis induction, we assessed caspase-3 activation in Z181 and Z119 cells exposed to increasing doses of canertinib for 18 hours. In both cell lines, there was significant activation of caspase-3 after treatment (Fig. 5C). Activation of caspase-3 leads to DNA fragmentation and cell death; therefore, PI staining followed by FACS analyses was performed after treatment to determine the subdiploid population, a marker for DNA fragmentation, a hallmark of apoptosis. The subdiploid population was elevated in both cell lines after treatment (Fig. 5D). Additionally, the Annexin-V positivity of Z119 cells treated with canertinib was increased (Fig. S1). Together, these results suggest that canertinib treatment resulted in apoptosis of Ph^+^ALL cells.

**Figure 6:**
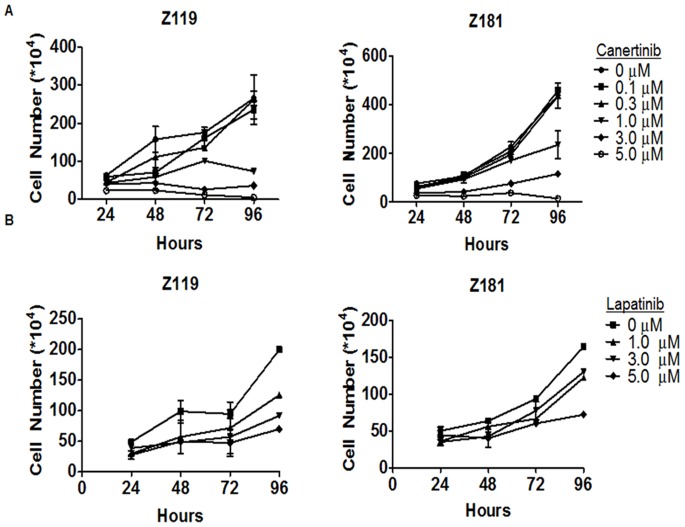
Decreased proliferation of ErbB2^+^Ph^+^ALL cells is induced by ErbB inhibition. Z119 and Z181 cells were treated with indicated doses of (A) canertinib or (B) lapatinib and allowed to proliferate for 96 hours. Cell viability and total cell number were determined every 24 hours by trypan blue exclusion. Points indicate the mean and SEM of viable cell numbers in at least three independent experiments.

To determine if inhibition of ErbB-dependent signaling was sufficient to abrogate proliferation of Ph^+^ALL cells, we measured the cell yield over 96-hours with increasing doses of canertinib (Fig. 6A). Canertinib effectively inhibited the proliferation of both Z119 and Z181 cells (Fig. 6A), yielding an IC_50_ of 0.78 µM and 1.18 µM, respectively. While canertinib has been utilized experimentally as a pan-ErbB inhibitor for a number of years, questions remain regarding its specificity [14]. Therefore, proliferation of Ph^+^ALL cell lines was measured after treatment with the more specific FDA approved ErbB1/ErbB2-directed TKI lapatinib. Much like canertinib, lapatinib inhibited the proliferation of Z119 and Z181 cells in a dose-dependent manner throughout the 96-hour period (Fig. 6B). These results suggest that intracellular targeting of the ErbB2 receptor is sufficient to inhibit proliferation of Ph^+^ALL cells.

### ErbB-family Kinase Inhibition Sensitizes ErbB2^+^Ph^+^ALL Cells to BCR/ABL-directed TKI

**Figure 7:**
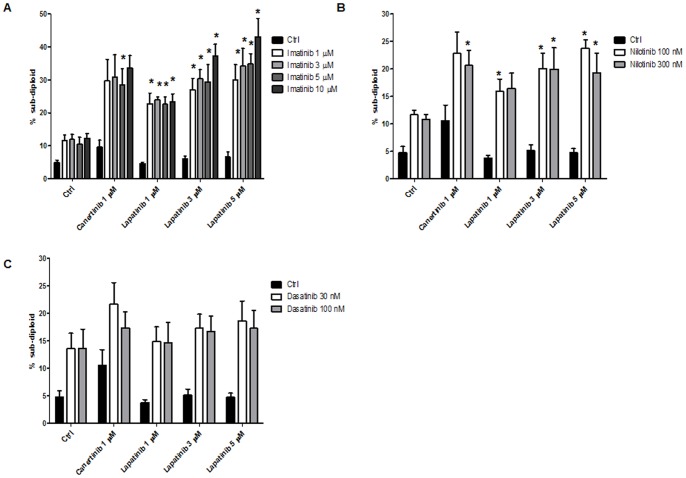
Small molecule ErbB2 inhibitors increase sensitivity to BCR/ABL-directed TKI. Z119 cells were treated with indicated doses canertinib or lapatinib and (A) imatinib, (B) nilotinib or (C) dasatinib for 72 hours. Cells were stained with PI and the subdiploid population was measured by flow cytometry. Bars indicate the mean and SEM of at least three independent experiments.

Since our data show that inhibition of ErbB signaling is effective in Ph^+^ALL, we explored its utility in the context of BCR/ABL inhibition. Z119 cells were treated for 72 hours with clinically relevant doses of BCR/ABL-directed TKI (Fig. 7A imatinib [15], Fig. 7B nilotinib [16], and Fig. 7C dasatinib [17]) and ErbB2-directed TKI (canertinib and lapatinib [18]) alone and in combination and the subdiploid population was measured. Combinations of either canertinib or lapatinib with imatinib and nilotinib produced significantly more cell death than single agents. The effects of dasatinib, a dual BCR/ABL-Src kinase inhibitor, were not significantly potentiated by canertinib or lapatinib. Together, these data suggest that inhibition of ErbB signaling increases the sensitivity of ErbB2^+^Ph^+^ALL cells to BCR/ABL-directed TKI.

## Discussion

Using specimens from 129 ALL patients, we have demonstrated that amplified ErbB2 expression and activation are seen in patients with Ph^+^ disease (Table 2 and Fig. 1). Additionally, inhibition of the ErbB signaling axis influences growth and survival signaling in ErbB2^+^Ph^+^ALL cells (Figs. 3–5). Specifically, canertinib, a pan-ErbB TKI, abrogates p70S6-kinase and S6-kinase phosphorylation, and perpetuates Bim expression, PARP cleavage, and caspase activation leading to apoptosis (Figs. 3–5). Relevant to development of a therapeutic strategy, we have found that either canertinib or the clinically available ErbB inhibitor lapatinib combined with BCR/ABL inhibitors resulted increased cell death of ErbB2^+^Ph^+^ALL cells (Fig. 7). Together, these results suggest that the combination of ErbB-directed TKI with currently used BCR/ABL targeted therapies may improve outcome in this aggressive disease.

Previous work has analyzed the expression of ErbB in B-lineage ALL [3], [4]; however, to date, there has been no delineation of subtypes of ALL that ErbB2 expression may be associated with. Our analysis of 129 ALL patient samples revealed that ErbB2 overexpression is seen in approximately one-third of ALL patients. While there are lineage specific changes in this number (B-ALL having ∼30%, while T-ALL ∼50%), these may be explained by variability in sample sizes for these groups. The implications of these differences remain to be seen. However, it is clear that ErbB2 is overexpressed and overactivated in the Ph^+^ALL subgroup. As stratification of Ph-status is an important prognostic indicator in this disease, this finding may provide important insight into the biology of this subgroup.

Two human-derived Ph^+^ALL cell lines, Z181 and Z119, both express properly localized and active ErbB2 (Fig. 2) and trace amounts of other ErbB family members (data not shown). Therefore, these cell lines are useful tools in the understanding of this subpopulation. Differences between these two cell lines have been characterized, including differential sensitivity to growth factors [5]. ErbB2 expression also differs between these cell lines. Z181 cells contain almost 50-fold more ErbB2 than Z119 cells, which may explain the higher IC_50_ value for ErbB TKIs in this cell line. Together with our results showing that ErbB2 is autophosphorylated in both cell lines (Fig. 2C), our findings indicate that a subset of Ph^+^ALL cells express active ErbB2.

In both Z181 and Z119 cells, canertinib treatment not only decreased ErbB2p; it also resulted in a significant decrease in the activation of signaling components downstream of mTOR including p70S6- and S6-kinases (Fig. 3B). S6-kinase, a component of the 40S ribosomal subunit, is involved in the regulation of cell size, proliferation, and metabolic homeostasis. It is phosphorylated by p70S6-kinase, a target of mTOR signaling. P70S6-kinase, nor its target S6-kinase, have previously been shown to be altered downstream of canertinib treatment. However, these data are congruent with published work suggesting that alterations of the mTOR pathway are required for ErbB2 inhibitors to carry out their anti-tumor effects in solid tumor systems [19]. Potentially more relevant to the work presented herein, elevations of mTOR signaling have been associated with decreased imatinib sensitivity in Ph^+^ALL [20].

RPPA analyses, validated by western blotting, also revealed that canertinib treatment elevated levels of the pro-apoptotic BCL-2 family member Bim (Figs. 3 and 5). Modulation of Bim has been noted in ALL by a variety of therapeutic agents [21], [22], suggesting an importance of this pathway to apoptosis within leukemia cells. Induction of Bim in our model cells coincided with elevated PARP cleavage seen by western blotting, which was not captured by RPPA analysis (Fig. 5B). This discrepancy may be due to the technical limitations of the validated cleaved-PARP antibody used for RPPA analysis. Regardless, activation of caspase-3 (Fig. 5C) and increased subdiploid staining (Fig. 5D) suggest that canertinib promotes apoptosis in ErbB2^+^Ph^+^ALL cells. Additionally, canertinib was sufficient to fully inhibit proliferation of Z119 and Z181 cells (Fig. 6A). To further address specificity for the ErbB2 pathway, clinically relevant doses of the ErbB1/2-specific TKI lapatinib were used which, much like canertinib, abrogated proliferation of ErbB2^+^Ph^+^ALL cell lines (Fig. 6B). Together these data suggest that clinically available ErbB-directed TKIs have a marked effect on ErbB2^+^Ph^+^ALL proliferation and survival, therefore they may be of use in the treatment of this disease.

Interestingly, ErbB2 expression has also been correlated with chemoresistance in ALL [4]. Recent data from breast cancer models suggest that inhibition of Abl kinase activity with imatinib results in increased sensitivity to lapatinib [23]. Our data suggest a similar relationship in Ph^+^ALL: ErbB inhibition with clinically achievable doses of lapatinib or canertinib sensitized ErbB2^+^Ph^+^ALL cells to treatment with BCR/ABL-directed TKI (Fig. 7A&B). Interestingly, the effects of dasatinib (Fig. 7C), a dual BCR/ABL-Src kinase inhibitor were not potentiated by combinations with ErbB2-directed TKI, suggesting that more specific inhibition of the BCR/ABL and ErbB2 pathways is desirable. As lapatinib and imatinib/nilotinib are FDA-approved for use in various cancer types, our data suggest a clinical opportunity for the personalization of therapy for the subset of Ph^+^ALL patients that exhibit ErbB2 expression.

## Supporting Information

Figure S1 Canertinib increases Annexin-V positivity of Z119 cells in a dose-dependent manner. Z119 cells were treated with canertinib for 24 or 48 hours at indicated doses then stained with FITC-Annexin-V and analyzed by flow cytometry. (Top panel) Representative histograms of staining at the maximal dose of canertinib at 24 and 48 hours. (Bottom panel) Percentage Annexin-V positive cells as measured by flow cytometry. * indicates p<0.05
(TIF)

Table S1 Antibodies used for RPPA. CS = Cell Signaling Technologies, SC = Santa Cruz, BD = BD Biosciences.
(XLSX)

Table S2 p-values for RPPA analysis. P-Values were calculated between untreated cells (0) and 0.1–5.0 µM canertinib treatment for each cell line. Values highlighted in yellow are statistically significant (p<0.05).
(XLSX)

## References

[1] Lee HJ, Thompson JE, Wang ES, Wetzler M (2011) Philadelphia chromosome-positive acute lymphoblastic leukemia: current treatment and future perspectives. Cancer 117: 1583–1594. 21472706 10.1002/cncr.25690

[2] Avivi I, Goldstone AH (2003) Bone marrow transplant in Ph+ ALL patients. Bone Marrow Transplant 31: 623–632. 12692601 10.1038/sj.bmt.1703899

[3] Buhring HJ, Sures I, Jallal B, Weiss FU, Busch FW, et al (1995) The receptor tyrosine kinase p185HER2 is expressed on a subset of B-lymphoid blasts from patients with acute lymphoblastic leukemia and chronic myelogenous leukemia. Blood 86: 1916–1923. 7544646

[4] Chevallier P, Robillard N, Wuilleme-Toumi S, Mechinaud F, Harousseau JL, et al (2004) Overexpression of Her2/neu is observed in one third of adult acute lymphoblastic leukemia patients and is associated with chemoresistance in these patients. Haematologica 89: 1399–1401. 15531467

[5] Estrov Z, Talpaz M, Zipf TF, Kantarjian HM, Ku S, et al (1996) Role of granulocyte-macrophage colony-stimulating factor in Philadelphia (Ph1)-positive acute lymphoblastic leukemia: studies on two newly established Ph1-positive acute lymphoblastic leukemia cell lines (Z-119 and Z-181). J Cell Physiol 166: 618–630. 8600166 10.1002/(SICI)1097-4652(199603)166:3<618::AID-JCP17>3.0.CO;2-2

[6] Kornblau SM, Womble M, Qiu YH, Jackson CE, Chen W, et al (2006) Simultaneous activation of multiple signal transduction pathways confers poor prognosis in acute myelogenous leukemia. Blood 108: 2358–2365. 16763210 10.1182/blood-2006-02-003475 PMC1895551

[7] Kornblau SM, Tibes R, Qiu YH, Chen W, Kantarjian HM, et al (2009) Functional proteomic profiling of AML predicts response and survival. Blood 113: 154–164. 18840713 10.1182/blood-2007-10-119438 PMC2951831

[8] Tibes R, Qiu Y, Lu Y, Hennessy B, Andreeff M, et al (2006) Reverse phase protein array: validation of a novel proteomic technology and utility for analysis of primary leukemia specimens and hematopoietic stem cells. Mol Cancer Ther 5: 2512–2521. 17041095 10.1158/1535-7163.MCT-06-0334

[9] Kornblau SM, Qui YH, Chen W, Chou P, Blau L, et al (2008) Proteomic Profiling of 150 Proteins in 511 Acute Myelogenous Leukemia (AML) Patient Samples Using Reverse Phase Proteins Arrays (RPPA) Reveals Recurrent Proteins Expression Signatures with Prognostic Implications [abstract]. Blood 112: 281–282.

[10] Hunyady B, Krempels K, Harta G, Mezey E (1996) Immunohistochemical signal amplification by catalyzed reporter deposition and its application in double immunostaining. J Histochem Cytochem 44: 1353–1362. 8985127 10.1177/44.12.8985127

[11] Hu J, He X, Baggerly KA, Coombes KR, Hennessy BT, et al (2007) Non-parametric quantification of protein lysate arrays. Bioinformatics 23: 1986–1994. 17599930 10.1093/bioinformatics/btm283

[12] Neeley ES, Kornblau SM, Coombes KR, Baggerly KA (2009) Variable slope normalization of reverse phase protein arrays. Bioinformatics 25: 1384–1389. 19336447 10.1093/bioinformatics/btp174 PMC3968550

[13] Rubin I, Yarden Y (2001) The basic biology of HER2. Ann Oncol 12 Suppl 1S3–8. 10.1093/annonc/12.suppl_1.s3 11521719

[14] Karaman MW, Herrgard S, Treiber DK, Gallant P, Atteridge CE, et al (2008) A quantitative analysis of kinase inhibitor selectivity. Nat Biotechnol 26: 127–132. 18183025 10.1038/nbt1358

[15] Peng B, Hayes M, Resta D, Racine-Poon A, Druker BJ, et al (2004) Pharmacokinetics and pharmacodynamics of imatinib in a phase I trial with chronic myeloid leukemia patients. J Clin Oncol 22: 935–942. 14990650 10.1200/JCO.2004.03.050

[16] Tanaka C, Yin OQ, Sethuraman V, Smith T, Wang X, et al (2010) Clinical pharmacokinetics of the BCR-ABL tyrosine kinase inhibitor nilotinib. Clin Pharmacol Ther 87: 197–203. 19924121 10.1038/clpt.2009.208

[17] Christopher LJ, Cui D, Wu C, Luo R, Manning JA, et al (2008) Metabolism and disposition of dasatinib after oral administration to humans. Drug Metab Dispos 36: 1357–1364. 18420784 10.1124/dmd.107.018267

[18] Burris HA, 3rd, Hurwitz HI, Dees EC, Dowlati A, Blackwell KL, et al (2005) Phase I safety, pharmacokinetics, and clinical activity study of lapatinib (GW572016), a reversible dual inhibitor of epidermal growth factor receptor tyrosine kinases, in heavily pretreated patients with metastatic carcinomas. J Clin Oncol 23: 5305–5313. 15955900 10.1200/JCO.2005.16.584

[19] Miller TW, Forbes JT, Shah C, Wyatt SK, Manning HC, et al (2009) Inhibition of mammalian target of rapamycin is required for optimal antitumor effect of HER2 inhibitors against HER2-overexpressing cancer cells. Clin Cancer Res 15: 7266–7276. 19934303 10.1158/1078-0432.CCR-09-1665 PMC2787848

[20] Xing H, Yang X, Liu T, Lin J, Chen X, et al (2012) The study of resistant mechanisms and reversal in an imatinib resistant Ph+ acute lymphoblastic leukemia cell line. Leuk Res 36: 509–513. 22285507 10.1016/j.leukres.2011.12.018

[21] Lu J, Quearry B, Harada H (2006) p38-MAP kinase activation followed by BIM induction is essential for glucocorticoid-induced apoptosis in lymphoblastic leukemia cells. FEBS Lett 580: 3539–3544. 16730715 10.1016/j.febslet.2006.05.031

[22] Rambal AA, Panaguiton ZL, Kramer L, Grant S, Harada H (2009) MEK inhibitors potentiate dexamethasone lethality in acute lymphoblastic leukemia cells through the pro-apoptotic molecule BIM. Leukemia 23: 1744–1754. 19404317 10.1038/leu.2009.80 PMC2761998

[23] Lo YH, Ho PC, Zhao H, Wang SC (2011) Inhibition of c-ABL sensitizes breast cancer cells to the dual ErbB receptor tyrosine kinase inhibitor lapatinib (GW572016). Anticancer Res 31: 789–795. 21498698

