# Supplementary figures and images for: Small Molecule ErbB Inhibitors Decrease Proliferative Signaling and Promote Apoptosis in Philadelphia Chromosome–Positive Acute Lymphoblastic Leukemia
Source: PLoS One. 2013 Aug 1;8(8):e70608. doi: 10.1371/journal.pone.0070608 (PMC3731286; doi:10.1371/journal.pone.0070608)

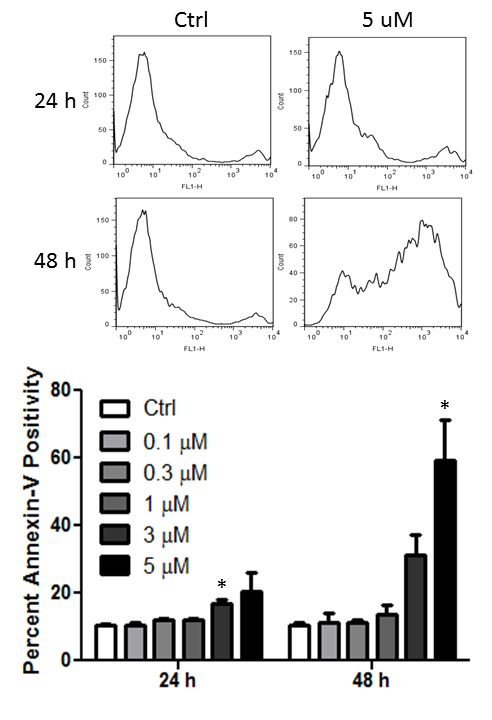

Supplement: Figure S1 — Z119 cells were treated with canertinib for 24 or 48 hours at indicated doses then stained with FITC-Annexin-V and analyzed by flow cytometry. (Top panel) Representative histograms of staining at the maximal dose of canertinib at 24 and 48 hours. (Bottom panel) Percentage Annexin-V positive cells as measured by flow cytometry. * indicates p<0.05 (TIF) [file pone.0070608.s001.tif]
